# Structural Insights into Novel 15-Prostaglandin Dehydrogenase Inhibitors

**DOI:** 10.3390/molecules26195903

**Published:** 2021-09-29

**Authors:** Prema L. Mallipeddi, Yongyou Zhang, Hongyun Li, Sanford D. Markowitz, Bruce Posner

**Affiliations:** 1 Department of Biochemistry, University of Texas Southwestern Medical Center, Dallas, TX 75390, USA; Prema.mallipeddi@UTSouthwestern.edu; 2 Department of Medicine, Case Western Reserve University, Cleveland, OH 44106, USA; yongyouzhang@xmu.edu.cn (Y.Z.); hxl922@case.edu (H.L.); sxm10@case.edu (S.D.M.); 3 Case Comprehensive Cancer Center, Case Western Reserve University, Cleveland, OH 44106, USA; 4 Simmons Cancer Center, University of Texas Southwestern Medical Center, Dallas, TX 75390, USA

**Keywords:** molecular docking, 15-PGDH, PGE2, inhibitor

## Abstract

We discovered SW033291 in a high throughput chemical screen aimed at identifying 15-prostaglandin dehydrogenase (15-PGDH) modulators. The compound exhibited inhibitory activity in in vitro biochemical and cell-based assays of 15-PGDH activity. We subsequently demonstrated that this compound, and several analogs thereof, are effective in in vivo mouse models of bone marrow transplant, colitis, and liver regeneration, where increased levels of PGE2 positively potentiate tissue regeneration. To better understand the binding of SW033291, we carried out docking studies for both the substrate, PGE2, and an inhibitor, SW033291, to 15-PGDH. Our models suggest similarities in the ways that PGE2 and SW033291 interact with key residues in the 15-PGDH-NAD+ complex. We carried out molecular dynamics simulations (MD) of SW033291 bound to this complex, in order to understand the dynamics of the binding interactions for this compound. The butyl side chain (including the sulfoxide) of SW033291 participates in crucial binding interactions that are similar to those observed for the C15-OH and the C16-C20 alkyl chain of PGE2. In addition, interactions with residues Ser138, Tyr151, and Gln148 play key roles in orienting and stabilizing SW033291 in the binding site and lead to enantioselectivity for the *R*-enantiomer. Finally, we compare the binding mode of (*R*)-S(O)-SW033291 with the binding interactions of published 15-PGDH inhibitors.

## 1. Introduction

15-PGDH belongs to a large family of short-chain alcohol dehydrogenases, which exhibit amino acid sequence homology ranging from 15–30% [1]. 15-PGDH controls intracellular levels of PGE2 by oxidizing the 15(*S*)-hydroxyl group on PGE2 to the corresponding keto group. Recent studies from our labs describe a role for inhibitors of 15-PGDH in potentiating tissue repair by increasing PGE2 levels [2,3,4,5]. Using a high throughput screening approach, we identified SW033291 (2-(butylsulfinyl)-4-phenyl-6-(thiophen-2-yl) thieno[2,3-b]pyridin-3-amine) as a novel agent that elevates PGE2 levels by inhibiting 15-PGDH with high affinity and selectivity [1]. However, it has been difficult to gain structural insights into the binding modes of SW033291 and its analogs due to challenges in obtaining co-crystals of 15-PGDH with inhibitor bound. We also wanted to compare the binding of SW033291 to that of PGE2, the natural substrate. Given that a crystal structure has not been published for the latter, we carried out a docking study of PGE2 and SW033291 (R and S enantiomers) to human 15-PGDH (PDBID: 2GDZ) [6]. As part of this study, we performed 30 ns molecular dynamics (MD) simulation studies of 15-PGDH with both enantiomers of this compound. Our model provides several insights into specific interactions between SW033291 and 15-PGDH and allows us to identify pharmacophore features that account for the tight-binding of (*R*)-S(O)-SW033291.

In addition to the initial discovery of SW033291, we carried out a successful medicinal chemistry program to further optimize this chemical hit to a lead compound with better physicochemical properties (e.g., solubility) and lower protein binding [3]. Antczak and coworkers generated a number of analogs that explored the structural requirements for potency and efficacy in both enzyme and cell-based inhibition assays for 15-PGDH. Furthermore, they demonstrated that the *R* enantiomer of SW033291 is ~300-fold more potent than the *S* enantiomer in the enzymatic assay. In this context, there are instances where our model is useful, namely, in predicting the enantiomeric selectivity and the activity of some analogs, which maintain high potency and selectivity while enhancing solubility and/or reducing protein binding. This is in line with other studies, where docking has been useful [7,8].

To summarize, this computational study provides potential binding modes for a novel PGDH inhibitor SW033291 and the substrate PGE2, supports a rationale for enantio-selectivity of (*R*)-S(O)-SW033291, compares the binding of (*R*)-S(O)-SW033291 to that of a subset of published 15-PGDH inhibitors, and suggests how (*R*)-S(O)-SW033291 is both potent and selective for 15-PGDH when compared to other members of the short chain dehydrogenase family of enzymes.

## 2. Results

In the absence of a co-crystal structure, molecular docking is a common tool used in drug discovery to identify potential ligand binding modes of small molecules to their target proteins [9]. Docking studies are usually performed by allowing the ligand to move within the defined protein binding site, while the protein structure is held rigid. In the case of SW033291 and PGE2, no one has determined co-crystal structures of these molecules with 15-PGDH, despite several attempts. In this context, we sought to carry out docking studies in order to gain insights into how the substrate (PGE2) and our inhibitor (SW033291) bind to this enzyme.

15-PGDH catalyzes the oxidation of the 15-hydroxyl group of PGE2 to 15-keto-PGE2. Niesen et al. published a crystal structure of human 15-PGDH in complex with NAD+ (PDBID 2GDZ) at 1.65 Ȧ resolution [6]. However, to date, no one has published an X-ray crystal structure of human 15-PGDH in complex with substrate PGE2 or a small molecule. The lack of success in generating structures of PGE2 and 15-PGDH is likely due to the fact that the former is labile and does not survive conditions necessary for crystallization. Niesen et al. docked three 15-PGDH inhibitors using Ser138 as an anchor point in their docking study. Their study concluded that the inhibitors interact with residues Ser138, Tyr151, Phe185, and Tyr217 in 15-PGDH. The most potent compound in their study, compound **13** (aka ML148), has an IC_50_ of 19 nM ([10]). The compound exhibited an AC50’s of 0.469 uM in PGE2 assay with A549 cells and 0.884 uM in LNCaP cells, with a maximal induction of PGE2 levels of 129% and 62%, respectively. No activity in animal models has been reported for this compound to date, which is probably due to its relatively short half-life (~7.3 min) in rat liver microsomes [10].

Although other inhibitors [6,11,12] have also been identified, SW033291 is the only chemical series that is potent (K_i,app_ of ~0.1 nM), selective, and active in in vivo mouse models [1]. We discovered this compound as a racemic mixture in a high throughput chemical screen using a cell-based reporter assay [1]. We demonstrated that SW033291 is a tight-binding inhibitor that appears to be non-competitive versus PGE2, based on steady-state kinetic analyses. It was also shown that this compound is active in several mouse models of tissue regeneration, including recovery from liver resection, rescue from dextran sulfate-induced colitis, and recovery following bone marrow transplant (BMT), where the compound accelerated regeneration of neutrophils by six days. Several other parameters for recovery from BMT were enhanced, including platelet count, hemoglobin levels, and accumulation of SKL cells in the bone marrow of recipient mice [1]. Although these studies showed that SW033291’s effects are specific to its inhibition of 15-PGDH, the binding mode of SW033291 to this enzyme has not been delineated experimentally or computationally.

PGE2 was previously docked into a 15-PGDH homology model by Hamza et al. [13]. The model was based on levodione reductase (33% sequence identity with 15-PGDH) and Tropinone Reductase (23% sequence identity with 15-PGDH). NAD+ binding was modeled by superimposition. 15-PGDH-NAD+ was visually analyzed for possible PGE2 binding pockets. PGE2 was subsequently docked into the modeled structure. However, the Hamza model is not consistent with respect to the 15-PGDH-NAD+ co-crystal structure published subsequently by Niesen et al. [6]. For example, in the Hamza model, the 15-OH of PGE2 is predicted to interact with Ser138 (1.78 Ȧ) and Gln148 (1.80 Ȧ). The Cα distance between the two residues is ~11.9 Ȧ in the co-crystal structure published later, making the Hamza model implausible in this respect [6]. Moreover, Hamza and co-workers predicted that Gly250 along with residues Val145, Ala146, Gly184, and Ile194 line PGE2 binding site. However, the co-crystal structure reveals Gly250 to be far away from key active site residues (e.g., distance between Cα of S138 and Gly250 is 13.92 Ȧ). Thus, these disconnects raise questions about this model, which was based on homologous proteins with less than 40% sequence identity. Hence, we felt that docking PGE2 against the 15-PGDH-NAD+ X-ray crystal structure was more appropriate and would help decipher structural insights into PGE2 interactions with 15-PGDH.

**Figure 1:**
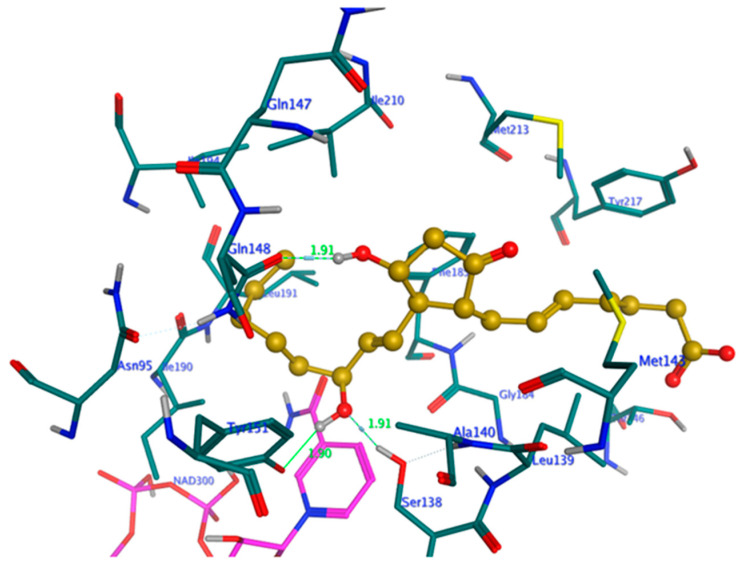
PGE2 docked pose showing H-bonding interactions Ser138, Tyr151, and Gln148. Also shown are residues within 3.5 angstroms of PGE2. The C2-C7 alkyl chain, as well as the C16-C20 alkyl chain of PGE2, are predominantly surrounded by hydrophobic residues. PGE2 is shown in gold.

As starting point, we aimed to identify a reasonable binding pose for PGE2 and to outline the crucial interactions that specify substrate binding to 15-PGDH. As a first step, we searched for all potential small molecule binding sites in the 15-PGDH-NAD+ X-ray crystal structure. For this task, we used Castp (Computed atlas of surface topography of proteins), an online binding site prediction tool developed by Tian et al. [14]. The crystal structure of human 15-PGDH (PDBID: 2GDZ) was submitted to the Castp server to identify potential ligand binding pockets. It is notable that a similar binding site analysis was not done previously by Niesen et al. in their study [6]. Our analysis revealed only one small molecule binding site with an area of 545.4 Ȧ^2^ and volume of 316.7 Ȧ^3^ (Supplementary Figure S1). As expected, the predicted binding site encompasses catalytically important residues Ser138 and Tyr151 as well as Gln148 (Figure 1).

### 2.1. Docked Pose of PGE2

In our docking studies of PGE2, we generated multiple conformations of this molecule (See Section 5 for details). The final docking model is shown in Figure 1. The model reveals that the 15-hydroxyl group exhibits hydrogen bonding interactions with Tyr151 as well as Ser138. Ser138 and Tyr151 are conserved residues of the SDR family [15]. Our docked model of PGE2 binding to 15-PGDH is consistent with structures of enzyme-substrate complexes for members of the short-chain dehydrogenase family, of which 15-PGDH is a member [16]. 7α-Hydroxysteroid dehydrogenase (7α-HSDH), a member of the SDR family, was co-crystallized with NAD+ and oxidized substrate 7-OXO-GCDCA (7-oxoglycochenodeoxycholic acid) [16,17]. The co-crystal structure of 7α-HSDH shows that the analogous active site residues, Tyr159 and Ser146, interact with the hydroxyl group of the substrate. Overlay of co-crystal structure of 7α-HSDH with our PGE2 docked 15-PGDH model further confirms this observation (Supplementary Figure S2). Hence, this experimental observation supports our docking model for PGE2 and 15-PGDH. Site-directed mutational studies with human 15-PGDH demonstrated that Ser138 and Tyr151 are required for 15-PGDH activity [15]. For example, mutation of Tyr151 of human 15-PGDH to Ala, Phe, or Ser resulted in a catalytically inactive enzyme [15]. It is important to note that mutating Tyr151 to Phe renders the mutant enzyme inactive, suggesting the hydroxyl group plays a key role in the catalytic mechanism of 15-PGDH. Similarly, mutation of Tyr152 in a homologous protein, 11-beta-hydroxysteroid dehydrogenase, to Phe or Ser rendered the mutant enzyme inactive [18]. Site-directed mutagenesis of Ser138 in human 15-PGDH to alanine also resulted in inactive enzyme [19]. Mutation of Ser146 to Ala or His also dramatically reduced activity in 7α-HSDH, indicating the importance of a hydrogen bonding interaction with the substrate to maintain an ideal angle for proton transfer [17]. The S138A mutation in the homologous protein 3β/17β-HSDH resulted in an inactive enzyme; however, mutating this residue to Thr retained activity in the mutant enzyme [20]. These studies demonstrate that the Ser138 sidechain’s hydrogen bonding interaction with PGE2 is essential for catalysis [20]. Thus, our docked model of PGE2 is consistent with structural and mutational studies, which indicate that these conserved active site residues play an important role in substrate binding and orientation for catalysis in SDR family members.

In our model for PGE2 docked with human 15-PGDH, the importance of Gln148 is also evident. The C11-OH of PGE2 hydrogen bonds with the side chain of Gln148 (hydrogen bonding distance is 2.1 Å). Site-directed mutagenesis studies on human 15-PGDH indicate Gln148 serves as a hydrogen bond acceptor for PGE2 [18,21,22,23,24]. Gln148 has also been identified to be important in positioning substrate for catalysis, based on ternary crystal structures of levodione reductase (The analogous residue is Gln162) [25] and tropinone reductase-II (The analogous residue is Glu156) [26]. Further, mutation of Gln148 to Ala148 renders 15-PGDH inactive. This is in contrast to the Gln148Glu, Gln148His, and Gln148Asn mutations, which are acceptable substitutions with marginal activity losses [27]. Although this residue is not strictly conserved in all SDR family members, our model agrees with biochemical studies for 15-PGDH, where this residue appears to play an important role. In summary, the PGE2 docked pose and the predicted interactions with key residues in 15-PGDH agree well with previous structural and biochemical studies.

Based on our model, we identified additional interactions that appear to be important for PGE2 binding (Figure 1). Residues Gly184 and Phe185 orient the middle ring of PGE2 via hydrophobic interactions. The C16-C20 alkyl chain of PGE2 fills in the pocket formed by predominantly hydrophobic residues, such as Asn95, Ile190, Leu191, Ile194, and Ile210 (hydrophobic surface area 28.6 Ȧ^2^). The C2-C7 alkyl chain of PGE2 extends between residues Tyr217, Thr246, Met213, Leu139, and Met143 and exhibits hydrophobic interactions with these residues (hydrophobic surface area 174.9 Ȧ^2^).

### 2.2. Docked Pose of SW033291

**Figure 2:**
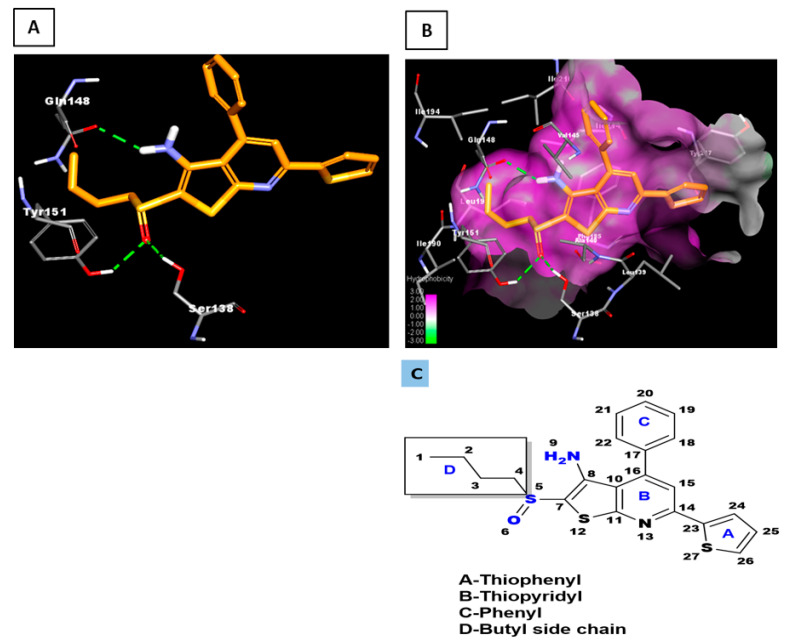
(**A**). Docked pose of (*R*)-S(O)-SW033291 showing hydrogen-bond interactions with residues Ser138, Gln148, and Tyr151. (*R*)-S(O)-SW033291 is colored gold while 15-PGDH is colored by atom. (**B**). Surface view of residues within 3.5 Ȧ of (*R*)-S(O)-SW033291 docked pose revealing binding site residues are mostly hydrophobic in nature. The hydrophobic binding surface is represented using the scale in the lower left hand corner of the figure. (**C**). (*R*)-S(O)-SW033291 showing atom numbering. Crucial pharmacophore features contributing towards activity are highlighted in blue.

In a fashion similar to our study of PGE2, we docked the *R*- and *S*-enantiomers of SW033291 to 15-PGDH at the site we identified using Castp (See Section 5). The docked model of the *R*-enantiomer is shown in Figure 2, with 15-PGDH represented as a ribbon with specific residues highlighted by stick representation (Figure 2A) and as a surface (Figure 2B). The latter shows that the surface of the binding site is largely hydrophobic in character, which complements the hydrophobic nature of the compound (cLogP ~5.6). Of importance, many of the interactions of the *R*-enantiomer with 15-PGDH are the same or similar to those we observed in our model for PGE2 binding (Figure 1 and vide infra).

**Figure 3:**
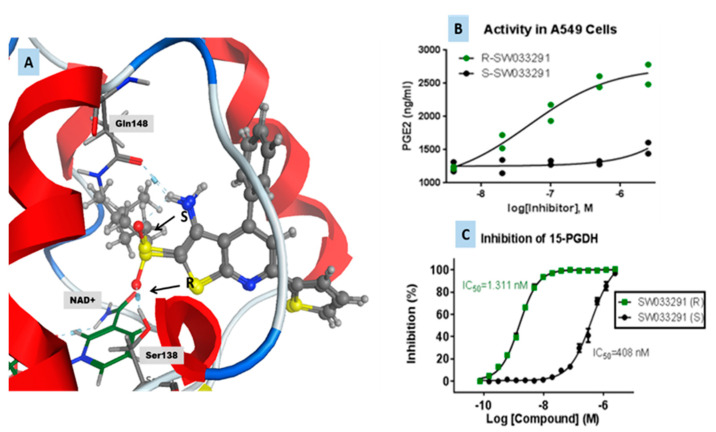
(**A**). Overlay of R versus S stereoisomers of SW033291. The oxygen of the sulfoxide in *R*-enantiomer exhibits hydrogen-bonding interactions with Ser138 and Tyr151. The *S*-stereoisomer prefers intramolecular hydrogen bonding. (**B**). PGE2 cell-based assay shows the (*R*)-S(O)-SW033291 is relatively more active than (*S*)-S(O) SW033291. (**C**). Percent inhibition (0–100%) of 15-PGDH enzyme activity of R versus S forms of SW033291.

Comparing our docked models of the *R*- and *S*-enantiomers, we can provide a rationale for the difference in potency observed between them in both biochemical and cell-based assays (Figure 3) [3]. We noticed a striking difference between these enantiomers that centers around interactions between the sulfoxide oxygen of the *R*-enantiomer and critical protein residues, which are absent in the binding mode of the *S*-enantiomer. The optimized pose of the *S*-enantiomer has an intramolecular hydrogen bonding interaction (2.2 Å) between the oxygen (O6) of the sulfoxide and the amino group (N9) of the thienopyridyl ring (Figure 2C and Figure 3A). In contrast, the distance between N9 and the sulfoxide oxygen is 4.6 Ȧ in *R*-enantiomer, thereby making such an interaction untenable. It is important to note that the criteria for hydrogen bonding includes both a donor-acceptor distance cut-off of 2.5 Å and a donor-hydrogen–acceptor angle ≥135°. In our model for the *R*-enantiomer, the sulfoxide oxygen forms hydrogen bonds with Ser138 and Tyr151 (Figure 2A and Figure 3A). The orientation of the sulfoxide oxygen in the *S*-enantiomer does not allow for these interactions (The distance between sulfoxide oxygen in *S*-enantiomer versus Tyr151 OH is 4.13 Ȧ and versus Ser138 OH is 3.75 Ȧ, both too long for a typical hydrogen bond.). Thus, relative to the *R*-enantiomer, the *S*-enantiomer loses hydrogen-bonding interactions with Ser138 and Tyr151 (Figure 3A).

**Figure 4:**
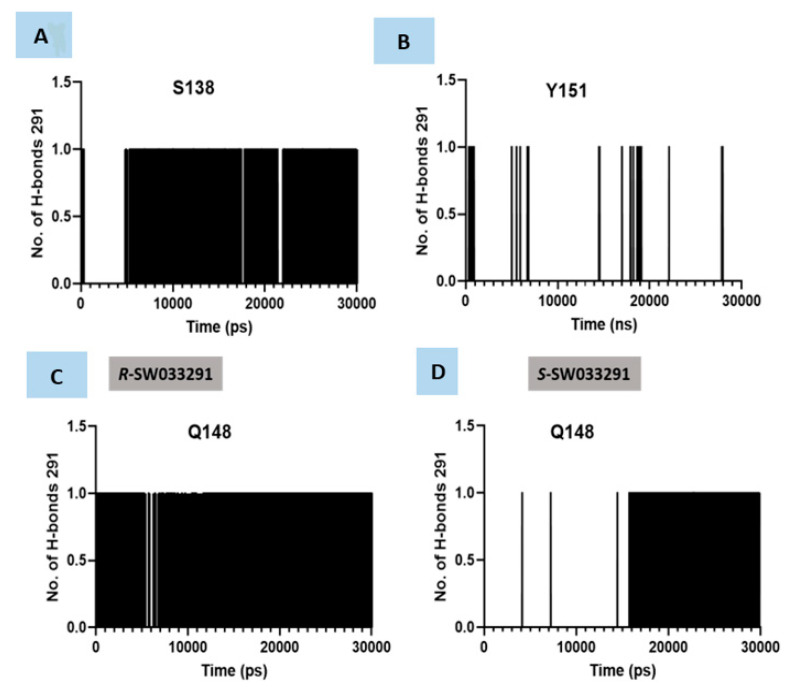
Ser138 consistently exhibits hydrogen bond interactions with (*R*)-SW033291 relative to Tyr151. Moreover, hydrogen-bond interactions with Gln148 are more prevalent in (*R*)-SW033291 vs. (*S*)-SW033291 across the 30 ns MD simulations. (**A**). Hydrogen bonding interactions of oxygen (O6) of sulfoxide (*R*)-X-SW033291 with side chain of Ser138 across the 30 ns MD simulations. (**B**). Hydrogen bonding interactions of oxygen (O6) of sulfoxide (*R*)-S(O)-SW033291 with Tyr151 side chain across the 30 ns MD simulations. (**C**). Hydrogen bond interactions of (*R*)-S(O)-SW033291 amino group with Q148 and (**D**). Hydrogen bond interactions of (*S*)-S(O)-SW033291 amino group with Q148.

Consistent with this result, we did not observe hydrogen bonding interactions in molecular dynamics simulations for Ser138 and Tyr151 and the sulfoxide of the *S*-enantiomer. On the other hand, the *R*-enantiomer exhibited hydrogen bonding interactions with Ser138 for 53.3% of the simulation. The distance between the sulfoxide (O6) of (*R*)-S(O)-SW033291 and Ser138 sidechain hydroxyl is within 2.5 Ȧ for 80% of the time. However, hydrogen bonding interactions were observed with Tyr151 only for 2.5% of the 30 ns MD simulations (Figure 4A,B). The distance between the sulfoxide (O6) of (*R*)-S(O)-SW033291 and Tyr151 side chain hydroxyl is within 2.5 Ȧ for 70.8% of the time; however, the angle criteria for effective hydrogen bonding is not met during most of the simulation (97.5% of the time).

The average donor-hydrogen–acceptor angle for the Ser138 OH and the oxygen of (*R*)-S(O)-SW033291 is 141.8°, while for Tyr151 OH, this angle is 89.9° in the MD simulations. It is important to note that the GROMACS donor-hydrogen–acceptor angle criteria for hydrogen bonding is ≥135 degrees. In general, the angle criteria for a strong hydrogen bond is closer to 180 degrees. Weak hydrogen bonds have bond angles less than 120 degrees, sometimes as low as 90 degrees [28]. Thus, based on the conformation of the 15-PGDH structure used in our model, we infer that the Tyr151 hydrogen bond is weak. Alternatively, a conformational change may occur in 15-PGDH that allows for a strong hydrogen bonding interaction to take place between these key residues (Ser138 and Tyr151) and (*R*)-S(O)-SW033291. This speculation is supported by the observed differences in potencies of the *R*- and *S*-enantiomers in the enzymatic 15-PGDH assay (Figure 3C).

In addition, the amino group (N9) of the *R*-enantiomer is free to interact with Gln148, thereby strengthening the binding interaction between this compound and 15-PGDH. In the *S*-enantiomer, (N9) can interact in an intramolecular fashion with the sulfoxide oxygen (O6), which could potentially weaken the hydrogen-bonding interaction with the Gln148. The distance between one of the hydrogens (H19) of the amino group and the carbonyl oxygen (OE1) of Gln148 in *R*-enantiomer is 2.2 Ȧ; while in *S*-enantiomer, it is 2.3 Ȧ. Consistent with this, molecular dynamics simulations reveal that the *R*-enantiomer exhibited a hydrogen bonding interaction with Gln148 for 63.1% of the time. In contrast, for the *S*-enantiomer, this interaction occurs for 31.4% of the same time frame (The distance between acceptor and hydrogen is less than or equal to 2.5 Å and the angle between donor-hydrogen–acceptor is greater than or equal to 135° (See Figure 4C,D). The distance between the Gln148 sidechain oxygen atom (OE1) and H19 of the amino group of SW033291 is within 2.5 Ȧ for 97.9% of the time for *R*-enantiomer, showing a high propensity for hydrogen bonding. For *S*-enantiomer, the distance is within 2.5 Ȧ for 30.5% of the time (Figure 4C,D). Consequently, the interaction of H19 of SW033291 with Gln148 appears to be weakened for the *S*-enantiomer. In summary, the cumulative effect of losing an interaction with Ser138 and Tyr151 and having a weakened interaction with Gln148 can potentially explain the weaker activity of *S*-enantiomer compared to the *R*-enantiomer, as observed by experimental studies (Figure 3B,C) [3].

Interestingly, our model of the *R*-enantiomer provides an explanation for the selectivity of SW033291 for 15-PGDH over other SDR family members. As discussed above, Gln148 appears to play an important role in binding this enantiomer of SW033291 to 15-PGDH. It is important to note that Gln148 is not a strictly conserved residue in the SDR family [19,27]. At this position, eight (8) out of 15 SDR family members have Gln, two (2) members have Glu, and three (3) have Asn and two (2) have His [27]. For example, the homologous protein Drosophila alcohol dehydrogenase (DADH) has a Val14 in place of Gln148. The residues Val14 and Leu95 (Leu95 corresponds to Asn95 in 15-PGDH) in DADH appear to be important for substrate specificity [29]. Likewise, when we looked at two other members of the SDR family members, 3-hydroxybutyrate dehydrogenase (BDH2) and 17-β-Hydroxysteroid dehydrogenase 10 (HSD17B10), we found that SW033291 could not be accommodated in their active sites. Overlay of the (*R*)-S(O)-SW033291 docked pose in the putative binding sites of these two proteins reveals potential clashes with non-homologous residues. The BDH2 binding site has Arg144 in place of Gln148 and an Arg (position 188) in place of Ile194 in 15-PGDH [30]. The sidechains of Arg144 and Arg188 project into the binding site and potentially block binding of (*R*)-S(O)-SW033291. Similarly, HSD17B10 has a Leu (position 259) in place of Lys249 (15-PGDH). The former sidechain projects into the region where (*R*)-S(O)-SW033291 would putatively bind in HSD17B10 [31]. Gln166 of HSD17B10 (equivalent to Gln148 of PGDH), is not in the right spatial orientation to have optimal interaction with (*R*)-S(O)-SW033291. In line with these observations, (*R*)-S(O)-SW033291 exhibited no activity in a thermal shift assay with BDH2 and HSD17B8 [1].

## 3. Discussion

Comparison of the Docked Poses of *R*-S(O)-SW033291 and PGE2.

**Figure 5:**
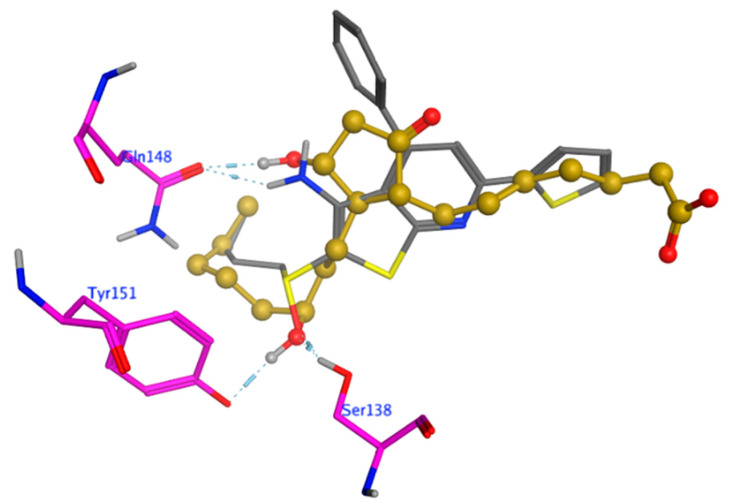
Overlay of the docked pose of (*R*)-S(O)-SW033291 (colored grey) vs. PGE2 (colored yellow) reveals differences between (*R*)-S(O)-SW033291 and PGE2.

As part of our study, we compared the docked pose of the *R*-enantiomer relative to that of PGE2. Overlay of the *R*-enantiomer of SW033291 and PGE2 highlights several similarities with respect to binding conformation, chemical features, and crucial interactions with residues Ser138 and Gln148 (Figure 5).

Specifically, the docked models show hydrogen bonding interactions between the sidechains of Ser138 and Tyr151 in 15-PGDH and the oxygen of sulfoxide in (*R*)-S(O)-SW033291 or the 15-OH of PGE2. Both (*R*)-S(O)-SW033291 and PGE2 also exhibit hydrogen-bond interactions with Gln148 through their oxygen atoms at the sulfoxide and C-15 carbons, respectively (Figure 1, Figure 2 and Figure 5). The butyl side chain of (*R*)-S(O)-SW033291 fits in a hydrophobic pocket (area 43.8 Ȧ^2^) lined by residues Tyr151, Ile190, Leu191, Ser193, Ile194, Ile210, and Ile214. PGE2 also has an alkyl chain (C16-C20) occupying a similar position with a hydrophobic surface area of 28.6 Ȧ^2^ (Figure 1 and Figure 2). Based on the similarity in binding conformation and interaction with crucial residues, we conclude that (*R*)-S(O)-SW033291 shares some of the critical protein interactions of the substrate PGE2.

It is tempting to conclude that (*R*)-S(O)-SW033291 might be mimicking PGE2 in its binding to 15-PGDH. However, the experimentally determined K_m_ for PGE2 against human PGDH is 3.4 µM [32], while the K_i_ of (*R*)-S(O)-SW033291 is 0.1 nM [1]. In order to explain this, we focused on the three (3) chemical features that could be contributing to the higher binding affinity of SW033291 (Figure 5). The thiophene group of (*R*)-S(O)-SW033291 is one of three molecular differences between PGE2 and (*R*)-S(O)-SW033291. This moiety exhibits Van der Waals and columbic interactions with Leu139, Gly184, Phe185, Tyr217, and Thr246. A C2-C7 alkyl chain of PGE2 occupies this region of 15-PGDH. The alkyl chain of PGE2 is relatively more flexible with two rotatable bonds and appears to have weaker interactions with 15-PGDH relative to the rigid thiophenyl group of (*R*)-S(O)-SW033291.

The cyclopentanone moiety of PGE2 and the thiopyridyl ring system of (*R*)-S(O)-SW033291 constitute the second major difference between these two molecules. The thiopyridyl moiety is an important anchoring point of (*R*)-S(O)-SW033291 for binding to 15-PGDH. It orients all the key features of this molecule, such as the sulfoxide-containing butyl chain and the pendant amino group (N-9), with key residues on 15-PGDH. The thiopyridyl moiety is bound in between Ala140, Val145, Phe185, and Leu191 of 15-PGDH. This moiety exhibits favorable stacking interactions with Phe185 and hydrophobic interactions with other residues (Ala140, Val145, and Leu191). PGE2 has a relatively weaker and smaller hydrophobic cyclopentanone group in a similar position, exhibiting interactions with Leu139, Val145, Phe185, and Ile214. Residue-wise interaction energy analysis [33] shows that the thiopyridyl group exhibits relatively more favorable interactions with Ala140 compared to PGE2 (SW033291, −4.79 Kcal/mol vs. PGE2, −1.67 Kcal/mol) and with Val145 (SW033291, −4.79 Kcal/mol vs. PGE2, −1.18 Kcal/mol). The cyclopentanone in PGE2 anchors the 11-OH and 15-OH chemical features that contribute towards binding 15-PGDH (Figure 5). It is important to note that the cyclopentanone is conformationally more flexible relative to the thienopyridyl ring system, and hence, the hydroxyls of PGE2 have relatively weak hydrogen bonding interactions with 15-PGDH. Residue-wise interaction energy analysis also shows interaction energies of the N9 amino group of (*R*)-S(O)-SW033291 with Gln148 are higher (−3.89 Kcal/mol) compared to −0.60 Kcal/mol for the C11 hydroxyl of PGE2. For Ser138, its interaction energy with sulfoxide oxygen is estimated to be −2.98 Kcal/mol compared to −1.00 Kcal/mol estimated for the C11 hydroxyl of PGE2.

The third difference between PGE2 and (*R*)-S(O)-SW033291 is the phenyl group at the 6 position of the thiopyridyl ring. PGE2 lacks a similar group at this region. This phenyl ring of (*R*)-S(O)-SW033291 is oriented towards hydrophobic residues: Val145, Ala146, Gln147, and His209. Residue-wise interaction energy analyses for the phenyl ring of SW033291 and keto group of cyclopentanone of PGE2 give the following binding energy comparisons: −1.69 Kcal/mol vs. −1.59 Kcal/mol with Ala146, respectively, and −0.08 Kcal/mol vs. −1.47 with Gln147, respectively. Thus, phenyl group does not seem to add much to the binding of SW033291 to 15-PGDH and this is consistent with the SAR observed by Anctzak et al. [3].

To summarize, (*R*)-S(O)-SW033291 shares key features with PGE2, and yet, it has some additional features, such as the thiophenyl moiety and the thienopyridyl ring, that appear to contribute towards tighter binding of this compound to 15-PGDH. Indeed, Antczak and co-workers synthesized and tested several analogs of (*R*)-S(O)-SW033291 and concluded the thienopyridyl ring and thiophenyl ring are crucial for the compound activity against the enzyme and in cells [3]. Interestingly, the pendant phenyl group of SW033291 is not required for inhibition of 15-PGDH enzymatic activity as measured in the biochemical assay, but it appears important for permeability in 15-PGDH cell-based activity. The overall structure (*R*)-S(O)-SW033291 is relatively rigid compared to PGE2, which seems to be a key factor contributing to more favorable binding interactions, as discussed in detail above. Thus, the (*R*)-S(O)-SW033291 structure appears to have a “lock and key” fit to the PGE2 binding site in human 15-PGDH that results in potent enzyme inhibition. This result from our model may seem at odds with the previous steady-state kinetic characterization of SW033291 as a non-competitive inhibitor. However, it must be borne in mind that SW033291 binds to 15-PGDH ~34,000-fold better than PGE2, thereby making PGE2 a very poor competitor for this binding site. Hence, in the steady state kinetics experiments carried out previously by our group, SW033921 appears to be non-competitive versus PGE2 over the concentration range that PGE2 is soluble (5–40 μM) [1].

We further compared the key interactions that (*R*)-S(O)-SW033291 (1, Table 1, IC_50_ = 1.5 nM) makes with 15-PGDH with those made by six (6) other published structures of 15-PGDH inhibitors [6,10,11,12,34] (Table 1).

Individual overlays of these 15-PGDH inhibitors with (*R*)-S(O)-SW033291 revealed similarities and significant differences in their respective binding modes. Compared with (*R*)-S(O)-SW033291, Compound **13** (ML148), Compound **14c**, and Compound **4a** have 12.7-fold, 8-fold, and 14.7-fold shifts, respectively, in inhibitory activity in the in vitro enzymatic assay with 15-PGDH (Table 1). Inspection of the interactions that Compound **13** (ML148) and Compound **14c** make with 15-PGDH shows that these compounds share several features with (*R*)-S(O)-SW033291. In particular, Compound **14c** and Compound **13** (ML148) have a hydrogen-bond acceptor that can interact with Ser138 and Tyr151. The positioning of that hydrogen-bond acceptor is facilitated by the fused ring system that is conserved in both molecules. Although there are idiosyncratic differences between these compounds and (*R*)-S(O)-SW033291, the overall shape and orientation of these key elements (hydrogen bond acceptor relative to the fused ring system) appear to be conserved, which is reflected in their IC_50_ values (all less than 25 nM). However, unlike (*R*)-S(O)-SW033291, they do not have a hydrogen bond donor to interact with Gln148, which could account for their lower affinity for 15-PGDH.

On the other hand, the Compound **4a** docked pose reveals hydrogen bonding with Gln148, also a key residue for the binding of (*R*)-S(O)-SW033291, and polar interactions with Ser138 and Tyr151 in our docked pose. The tetrazole ring is close enough to have hydrogen bond interactions with these residues [10]. However, we note that Compound **4a** contains a PAINS sub-structure with an unstable dimethyl pyrrole, which could react with residues on 15-PGDH [35]. Subsequent to binding, the covalent modification of 15-PGDH by Compound **4a** could skew estimates of potency based on a reversible binding model. This possibility should be further explored. Of importance, Compound 4a lacks a fused rigid central ring system like those of Compound **14c** (benzimidazole), Compound **13** (ML148) (benzimidazole), and (*R*)-S(O)-SW033291 (thiopyridyl ring system). These fused ring systems appear to anchor other key features in appropriate positions for optimal interactions with 15-PGDH (e.g., interactions with Ser138 and Tyr151). Additionally, the fused ring systems appear to favorably complement the hydrophobic binding pocket of 15-PGDH.

The Compound **3** described by Wu et al. [12] lacks a hydrogen bond acceptor to interact with Ser138 and Tyr151 and a hydrogen bond donor to interact with Gln148. The compound also lacks a fused central ring system. Docking analysis shows a backbone hydrogen bonding interaction between the ‘oxy’ group in Compound **3** and Ala140 (Supplementary Table S1). Overall, the lack of a central, fused ring system and appropriately positioned hydrogen bond groups on that scaffold may account for relatively weak binding of Compound **3**, compared to (*R*)-S(O)-SW033291. Further, Compound **3** has a alkylidenethiazolone moiety, which could interfere with assays and covalently modify 15-PGDH due to the reactive α,β unsaturated carbonyl groups [35]. This may account for its relatively high potency (IC_50_ ~8 nM) in the biochemical assay for 15-PGDH activity.

Docked poses of L-Oreal 1 and L-Oreal 2 compounds exhibit no hydrogen bond interaction with Ser138, Tyr151, or Gln148. Docking analysis reveals that L-Oreal 1 interactions with 15-PGDH are predominantly hydrophobic. L-Oreal 2 compound has a tetrazole amino group that exhibits hydrogen bonding interactions with Met143. The L-Oreal 1 and L-Oreal 2 compounds exhibit weak arene-H interaction with the amino of Gln148. Thus, these compounds exhibit a different mode of binding compared to (*R*)-S(O)-SW033291 and display a much weaker inhibition of 15-PGDH.

To summarize, the (*R*)-S(O)-SW033291 may be defined using a four feature pharmacophore (Supplementary Table S1). Feature 1 is a hydrogen bond acceptor to interact with Ser138 and Tyr151. Feature 2 is a hydrogen bond donor to interact with Gln148 and feature 3 is a fused ring system that serves as a scaffold to hold features 1 and 2 in proper orientation to interact with Ser138, Tyr151, and Gln148, in addition to exhibiting hydrophobic interactions with key residues on 15-PGDH. While the three key features described here appear to be essential for the high-affinity binding of (*R*)-S(O)-SW033291 to 15-PGDH, the phenyl ring feature may be defined as a 4th hydrophobic feature. As mentioned previously, the phenyl ring of (*R*)-S(O)-SW033291 is not required for the inhibition of 15-PGDH in vitro, but it appears important for the cell-based activity [3]. Overlay of pharmacophore features of SW033291 with those of other published inhibitors reveals a lack of some of these features in the latter molecules (Supplementary Table S1).

**Scheme 1:**
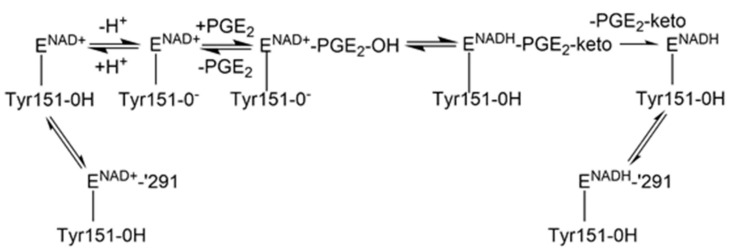
Proposed Mechanism for SW033291 Binding.

While docking and MD simulations provide structural insights into the binding of (*R*)-S(O)-SW033291 to 15-PGDH, the mode of binding will need to be further explored by structural and mutational studies. More specifically, modelling cannot address if (*R*)-S(O)-SW033291 is a transition state analog or simply a inhibitor of a ground state conformation of 15-PGDH. That said, a reductive analysis of the PGE2 and (*R*)-S(O)-SW033291 docked poses and previous experimental studies suggests a possible mechanism for the catalytic activity of 15-PGDH for the former and inhibition of 15-PGDH by the latter (Scheme 1). Structure studies showed that the hydroxyl group of substrate binds between residues Tyr151 and Ser138 [16,36,37,38]. A kinetic, pH dependence study by McKinley-McKee et al. with Drosophila alcohol dehydrogenase suggests that Tyr151 in 15-PGDH is in equilibrium between its protonated and deprotonated forms prior to substrate binding [39]. Once the substrate binds to the 15-PGHD-NAD+ complex, the hydride transfer from substrate to NAD+ occurs readily from the deprotonated form of the enzyme. Chen et al. [23] and Obeid and White [18] suggested a role of deprotonated Tyr in abstracting a proton from the substrate, which facilitates transfer of hydride to NAD+. Based on these studies and our analysis presented here, we and others have considered a similar mechanism for catalysis for 15-PGDH with its substrate PGE2 [6]. First, binding of NAD+ promotes deprotonation of Tyr151. After the substrate PGE2 binds, the deprotonated form of Tyr151 facilitates the hydride transfer from PGE2 to NAD+ by deprotonating the PGE2’s C15-OH. Based on thermal shift data, we found that (*R*)-S(O)-SW033291 appears to bind equally well to the 15-PGDH-NAD+ and 15-PGDH-NADH complexes with an increase in T_m_ of ~20 °C in both cases, relative to the cofactor–enzyme complex alone (Supplementary Table S2). For the deprotonated state of Tyr151 in the 15-PGDH-NAD+ complex, our model suggests a repulsive interaction between the sulfoxide group of (*R*)-S(O)-SW033291 and the negatively charged Tyr151 hydroxyl, thus making it unlikely that the inhibitor binds tightly to this state of the complex. Based on our model and biophysical data, we speculate that (*R*)-S(O)-SW033291 binds preferentially to the enzyme–cofactor complexes where Tyr151 is protonated. Interestingly, this implies that (*R*)-S(O)-SW033291 binds to the protonated form of Tyr151 in 15-PGDH-NAD+, thereby stabilizing non-productive ground-state complex and reducing the amount of enzyme that can facilitate proton transfer (Scheme 1). Of note, this mode of inhibition contrasts with that proposed for compounds **13** and **4a** [6], where thermal shift data suggests that these compounds preferentially bind to the 15-PGDH-NADH complex, where Tyr151 protonation is presumed to be favored. More mechanistic work and structural studies will need to be done to substantiate this mechanism for (*R*)-S(O)-SW033291 inhibition.

## 4. Conclusions

In the absence of a crystal structure, docking and molecular dynamics are useful tools to gain insights into the binding modes of small molecules to proteins. (*R*)-S(O)-SW033291 is a novel 15-PGDH inhibitor identified in a high throughput screen of a chemically diverse, small molecule library. To gain insights into the binding mechanism of (*R*)-S(O)-SW033291, we carried out docking studies for this compound and the 15-PGDH substrate, PGE2. The PGE2 and (*R*)-S(O)-SW033291 docked poses reveal key interactions with residues Ser138, Try151, and Gln148 on 15-PGDH. In biochemical studies, others have shown that these interactions are essential in 15-PGDH and analogous proteins. Of importance, hydrophobic interactions with residues Leu139, Ala140, Val145, Gly184, Phe185, Ile190, Leu191, Ser193, Ile194, Ile210, Ile214, Tyr217, and Thr246 further contribute towards the favorable binding of (*R*)-S(O)-SW033291. The vicinity of 15-OH of PGE2 in the docked model agrees well with a proposed mechanism of hydride transfer to NAD+. Overlay analysis of the PGE2 and the (*R*)-S(O)-SW033291 docked poses reveal similarities in chemical features between the substrate and the inhibitor. In particular, the oxygen of sulfoxide occupies a position similar to the 15-OH of PGE2, suggesting that the sulfoxide group of (*R*)-S(O)-SW033291 plays a crucial role. However, (*R*)-S(O)-SW033291 binds to human 15-PGDH approximately 34,000-fold more tightly than the substrate PGE2, assuming K_m_~K_d_ for PGE2 binding to 15-PGDH (PGE_2_ K_m_ ~3.4 μM; (*R*)-S(O)-SW033291 K_i_ ~0.1 nM). (*R*)-S(O)-SW033291 is also relatively more potent than other 15-PGDH inhibitors and 320-fold more so than the *S*-enantiomer. These differences in binding affinity underscore notable structural differences between (*R*)-S(O)-SW033291, PGE2, and other known PGDH inhibitors. The interaction of the amino (N9) group of (*R*)-S(O)-SW033291 with Gln148 of 15-PGDH is one of the major differences between the inhibitors discussed here and (*R*)-S(O)-SW033291. Moreover, both PGE2 and three of the 15-PGDH inhibitors that we considered here lack a fused aromatic ring system like the thiopyridyl ring in (*R*)-S(O)-SW033291, which serves to anchor this inhibitor in 15-PGDH’s PGE2 binding site. Thus, three of the four pharmacophore features—the hydrogen bond donor interaction with Gln148, the hydrogen bond acceptor interaction with Tyr151 and Ser138, and a rigid aromatic central thiopyridine ring system—appear to account for the relatively tight binding of (*R*)-S(O)-SW033291 to 15-PGDH compared to PGE2 and some of the PGDH inhibitors that we considered here. (*R*)-S(O)-SW033291 represents a unique chemical structure that contains several key features for optimal binding to 15-PGDH. Molecular dynamics simulations support the differences in binding modes of the *R*- and *S*-enantiomers of SW033291 identified by our model and may explain the large difference in their affinity for 15-PGDH. Thus, the model presented in this study appears to be reasonable and well supported. Of significance, this is the first structure-based modeling study on (*R*)-S(O)-SW033291, the only 15-PGDH inhibitor to exhibit high potency (K_i_ ~0.1) inhibition of 15-PGDH and in vivo activity in tissue regeneration models, where 15-PGDH appears to play a key regulatory role. Ultimately, binding of (*R*)-S(O)-SW033291 to the predicted binding site will need to be experimentally verified. To this end, structural studies of the (*R*)-S(O)-SW033291-(15-PGDH) complex are currently in progress.

## 5. Materials and Methods

### 5.1. Binding Site Prediction

PDB coordinates of 15-PGDH in complex with NAD+ (2GDZ) were submitted to Castp server to identify potential binding pockets [14]. Castp analysis of binding pocket reveals binding site with an area of 581.6 Ȧ^2^ and volume of 331.1 Ȧ^3^.

### 5.2. Docking

Crystal structure of 15-PGDH in complex with NAD+ (PDBID 2GDZ) at 1.65 Ȧ resolution was obtained from Protein Data Bank. The Protein Preparation Wizard in the Schrodinger Maestro v11.9.011 release 2019-1 (Schrodinger LLC, New York, NY, USA) was used to prepare protein for docking. The protein preparation includes addition of hydrogens and step-wise energy minimization using OPLS3e forcefield. PGE2, SW033291 (*R* and *S* enantiomers) and other analogs were prepared using the LigPrep module using the OPLS3e force field. This was followed by the generation of conformations in MOE v2018.01 (Chemical Computing Group, Montréal, QC, Canada). The conformation Limit was set to 200 and the LowModeMD method in MOE was used. The binding site predicted by Castp [14] was used to define Grid box and included crucial binding site residues such as Ser138, Tyr151 and Gln148. Atoms of the protein are held rigid while ligand atoms were allowed to move. Subsequently, the conformations were docked against 15-PGDH using Glide Schrodinger release 2019-1 (Schrodinger LLC, New York, NY, USA) standard precision (SP) method. Protein was treated rigidly while ligand was allowed to move flexibly during docking calculations. Docking protocol includes a systematic search to position ligand optimally within the defined binding site. Each of the docked complexes was energy minimized and the lowest energy poses were reported. Results were visually analyzed using Maestro v11.9.011, Discovery Studio Visualizer v2020 [40] and MOE2019.01 [41].

### 5.3. Molecular Dynamics

Molecular dynamics simulations were performed against R and S enantiomers of SW033291 using GROMACS 5.1.2 employing the GROMOS96 54A7 force field [42]. Ligand Topologies were generated using the ATB server [43]. For each simulation, the protein–ligand complex was immersed in a cubic box with at least 1.0 nm of water between the complex and edge of box. Termini of the protein were charged, system is neutralized and simulations were carried out using periodic boundary conditions. The system was equilibrated using NVT and NPT methods using reference temperature of 300 K. Particle Mesh Ewald (PME) methods were used to compute long range electrostatic interactions. This was followed by MD simulation for 30 ns on each system. All simulations were performed using Texas Advanced Computer Center (TACC) resources. Simulations were analyzed for root mean square deviation (RMSD), potential energy, hydrogen-bonding interactions using rms, hbond and energy scripts, respectively, from GROMACS [42]. For hydrogen bond interactions, the donor–acceptor distance cut-off of 2.5 Å and a donor-hydrogen–acceptor angle ≥ 135° were applied.

### 5.4. In Vitro Biochemical and Cell-Based Assays

The in vitro (enzymatic reaction) and the PGE2 cell-based assay for 15-PGDH activity were conducted as described previously [1].

## Figures and Tables

**Table 1:** Published structures of PGDH inhibitors.

ID	Structure	IC_50_ (nM)
SW033291 [1]	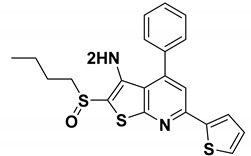	1.5
Compound **14c** [10]	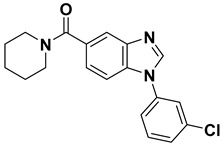	12
Compound **3** [12]	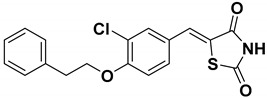	8
Compound **4a** [6,10]	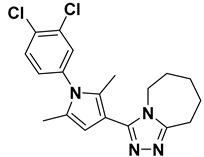	22
Compound **13** (aka ML148) [6,10]	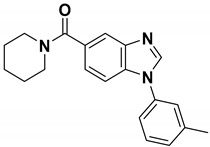	19
LOreal-1 [34]	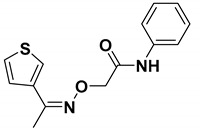	6000
LOreal-2 [11]	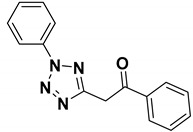	>6000

## Data Availability

Not applicable.

## Supplementary Materials

Supplementary Figure S1: Castp predicted binding site is shown in surface representation in green and PGDH is shown in gray in ribbon style. The predicted binding site has an area of 545.4 Ȧ^2^ and volume of 316.7 Ȧ^3^. Supplementary Figure S2: Overlay of 7α-HSDH co-crystal structure over the 15-PGDH-NAD+-PGE2 docked model. 15-OH of PGE2 docked model exhibits hydrogen-bond interactions with conserved residues Ser138 and Tyr151 similar to 7-OXO-GCDCA substrate in 7α-HSDH co-crystal structure. 7α-HSDH residues and substrate 7-OXO-GCDCA is colored teal while 15-PGDH residues are colored green. PGE2 is colored gold. Supplementary Table S1: Docked poses of published PGDH inhibitors. Supplementary Table S2: Thermal shift data showing (*R*)-S(O)-SW033291 binding to 15-PGDH-NAD+ and 15-PGDH NADH complexes.
