# Supplementary material for: Structural Insights into Novel 15-Prostaglandin Dehydrogenase Inhibitors
Source: Molecules. 2021 Sep 29;26(19):5903. doi: 10.3390/molecules26195903 (PMC8512612; doi:10.3390/molecules26195903)
Supplement: Supplementary file 1 [file molecules-26-05903-s001.zip › molecules-1394094-supplementary.pdf]

Supplementary Figure S1. Castp predicted binding site is shown in surface representation in green and PGDH is shown in gray in ribbon style. The predicted binding site has an area of 545.4 $Å^2$ and volume of 316.7 $Å^3$.

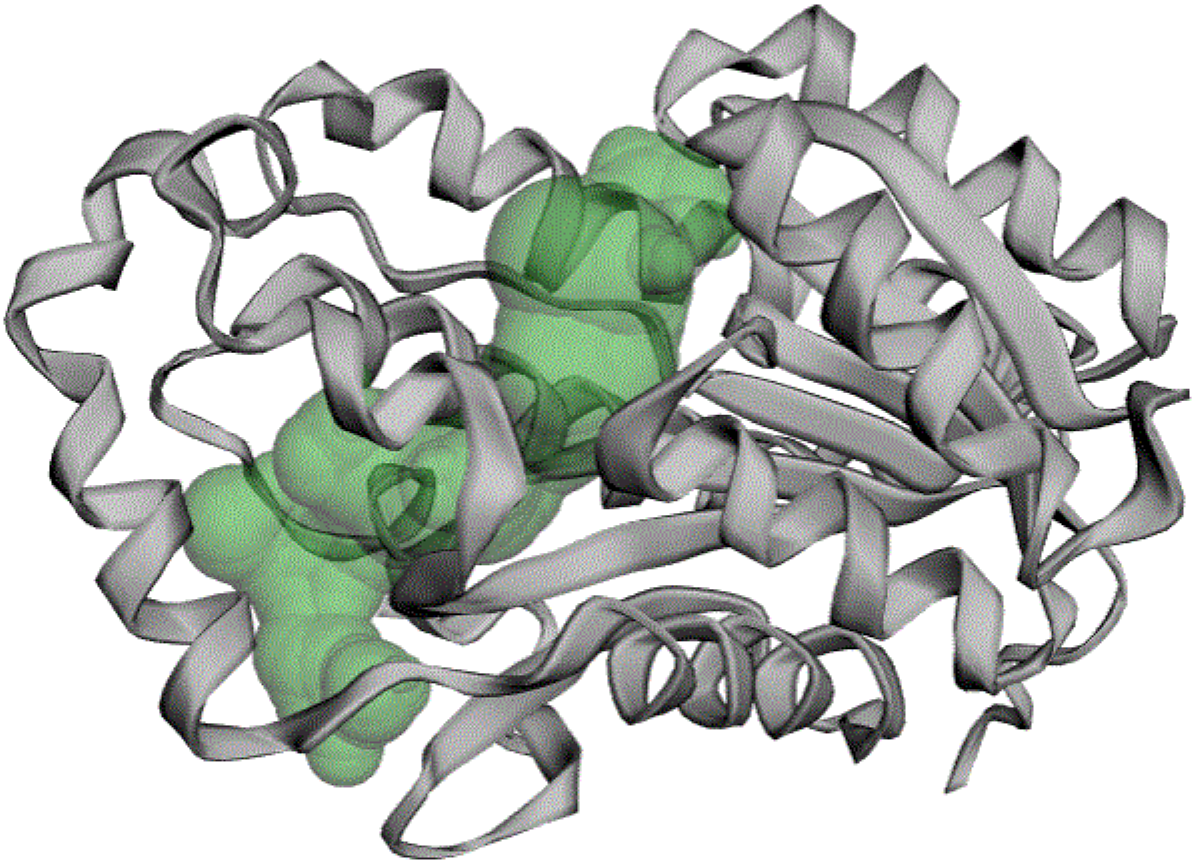

Supplementary Figure S2. Overlay of 7α-HSDH co-crystal structure over the 15-PGDH-NAD+-PGE2 docked model. 15-OH of PGE2 docked model exhibits hydrogen-bond interactions with conserved residues Ser138 and Tyr151 similar to 7-OXO-GCDCA substrate in 7α-HSDH co-crystal structure. 7α-HSDH residues and substrate 7-OXO-GCDCA is colored teal while 15-PGDH residues are colored green. PGE2 is colored gold.

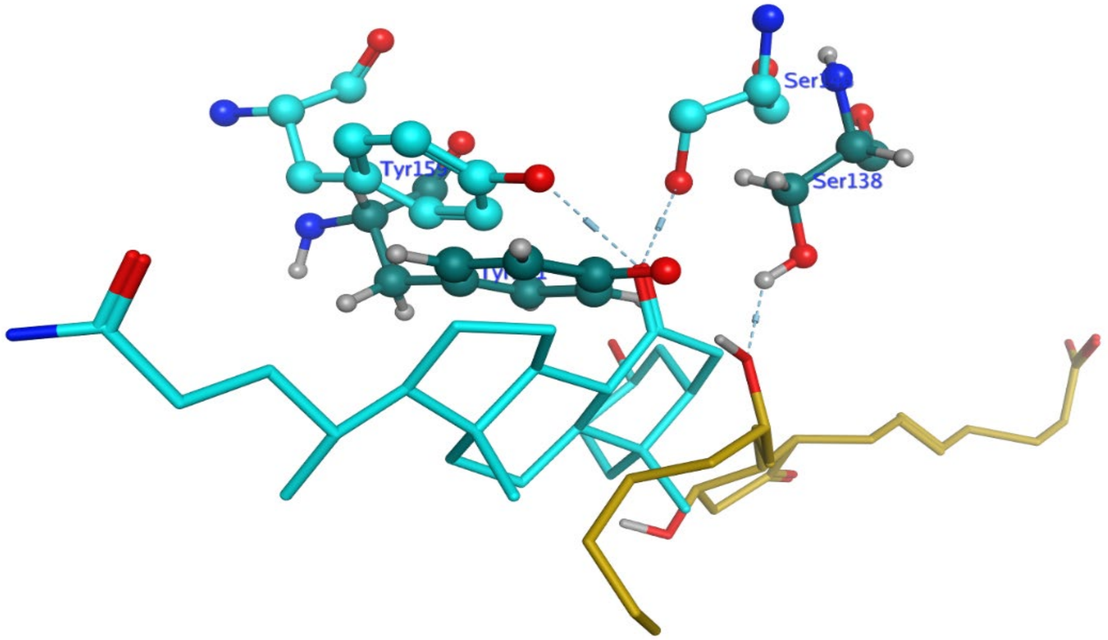

Supplementary Table S1. Docked poses of published PGDH inhibitors.

ID	Docked pose
Compound 14c [10]	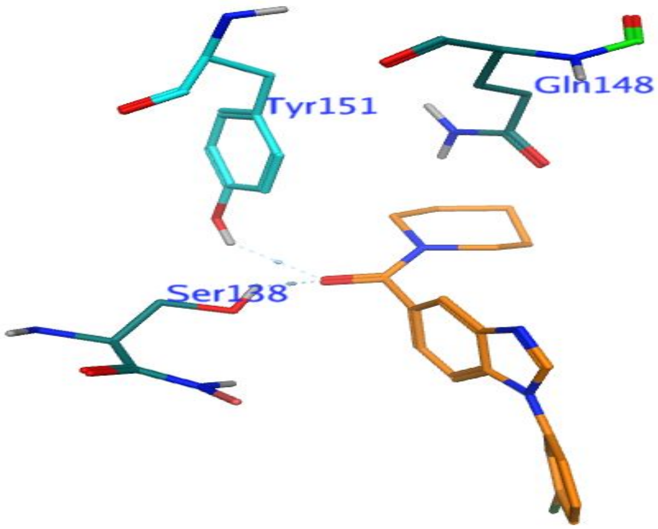
Compound 4a [6, 10]	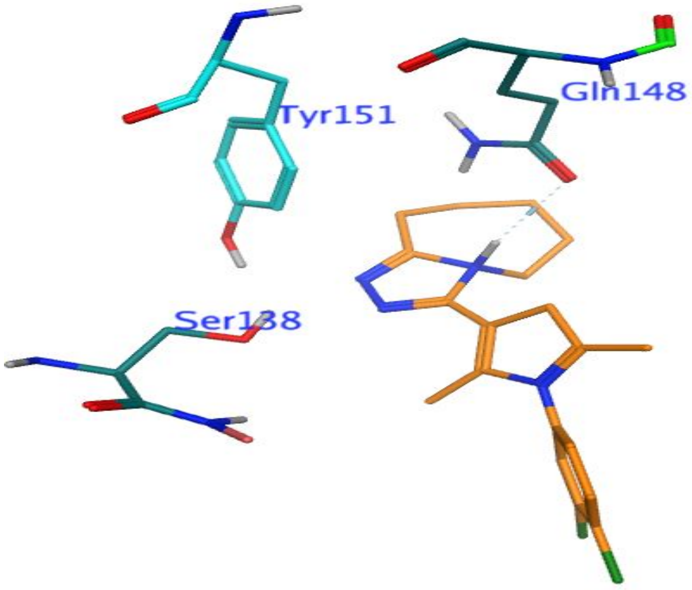

Compound 13 (aka ML148) [6, 10]	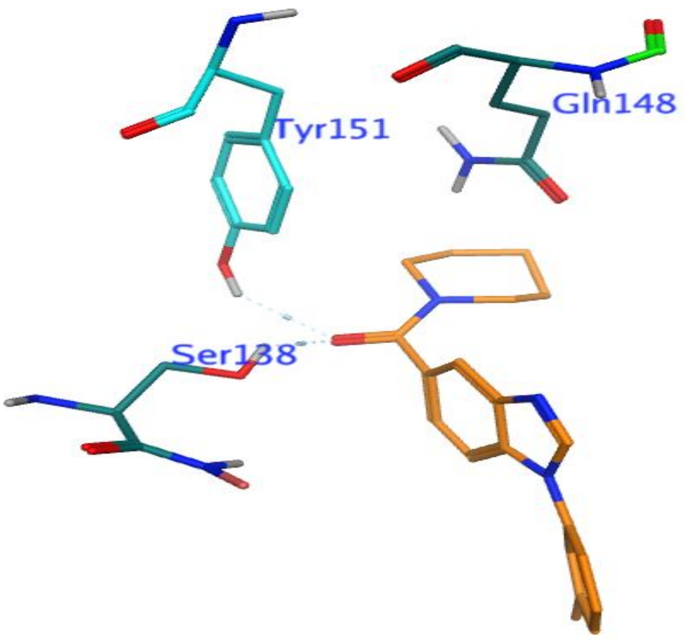
Compound 3 [12]	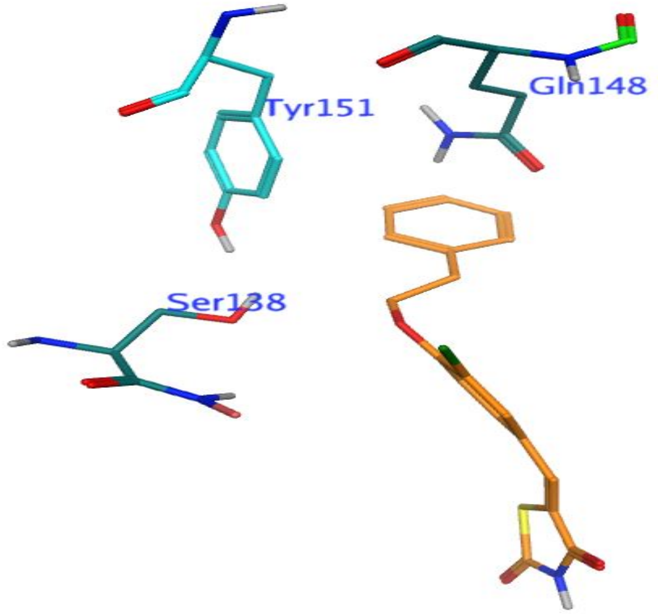

L-Oreal-1[35]	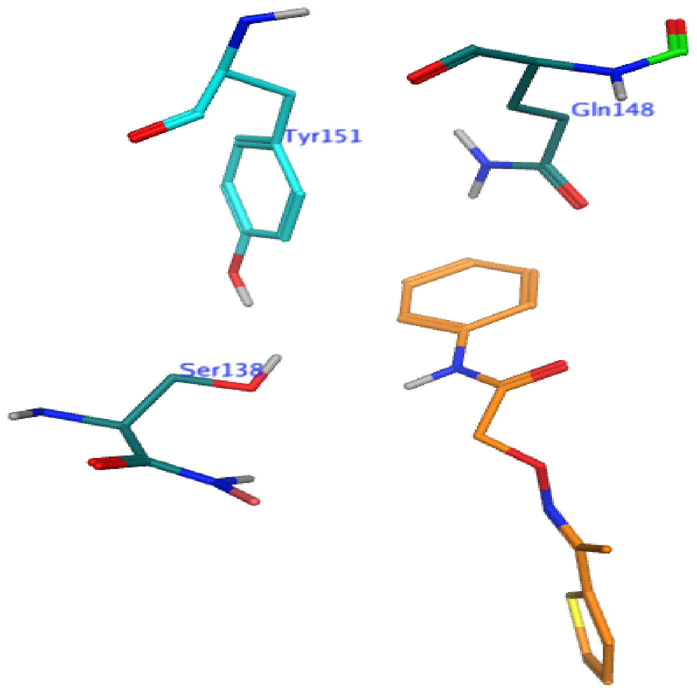
L-Oreal-2 [11]	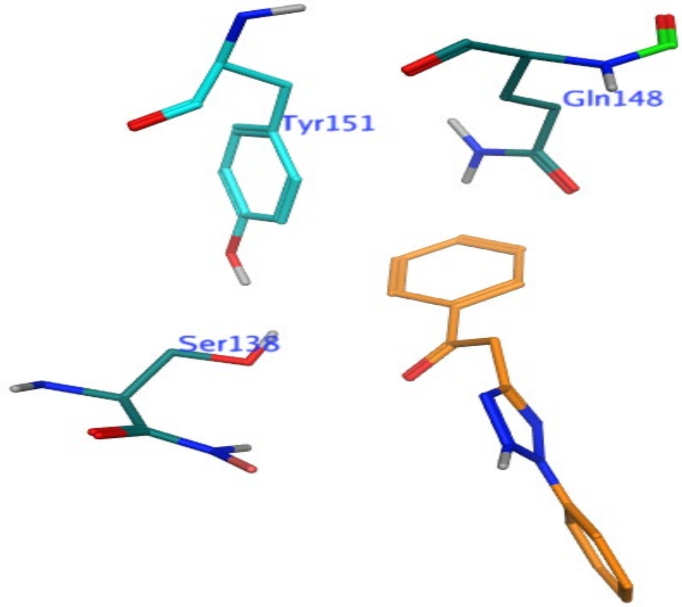

Supplementary Table S2. Thermal shift data showing (*R*)-S(O)-SW033291 binding to 15-PGDH-NAD+ and 15-PGDH NADH complexes.

Complex	Tm (ºC)
15-PGDH	44
15-PGDH/NAD(+)	45.5
15-PGDH/NADH	51
15-PGDH/NAD(+)/(*R*)-S(O)-SW033291	65.5
15-PGDH/NADH/(*R*)-S(O)-SW033291	72.5
